# Tumor Microenvironment Stimuli‐Responsive Single‐NIR‐Laser Activated Synergistic Phototherapy for Hypoxic Cancer by Perylene Functionalized Dual‐Targeted Upconversion Nanoparticles

**DOI:** 10.1002/advs.202203292

**Published:** 2022-08-28

**Authors:** Xiuna Jia, Deming Liu, Cong Yu, Niu Niu, Dan Li, Jin Wang, Erkang Wang

**Affiliations:** ^1^ State Key Laboratory of Electroanalytical Chemistry Changchun Institute of Applied Chemistry Chinese Academy of Sciences Changchun Jilin 130022 P. R. China; ^2^ State Key Laboratory of Luminescence and Applications Changchun Institute of Optics Fine Mechanics and Physics Chinese Academy of Sciences Changchun Jilin 130033 P. R. China; ^3^ Department of Chemistry and Physics State University of New York at Stony Brook New York 11794‐3400 USA; ^4^ College of Chemistry Jilin University Changchun Jilin 130012 P. R. China

**Keywords:** antihypoxic cancer, dual‐targeted nanosystem, photothermal–photodynamic synergistic therapy, single‐NIR‐laser trigger, TME‐responsive

## Abstract

Although synergistic therapy has shown great promise for effective treatment of cancer, the unsatisfactory therapeutic efficacy of photothermal therapy/photodynamic therapy is resulted from the absorption wavelength mismatch, tumor hypoxia, photosensitizer leakage, and inability in intelligent on‐demand activation. Herein, based on the characteristics of tumor microenvironment (TME), such as the slight acidity, hypoxia, and overexpression of H_2_O_2_, a TME stimuli‐responsive and dual‐targeted composite nanoplatform (UCTTD‐PC4) is strategically explored by coating a tannic acid (TA)/Fe^3+^ nanofilm with good biocompatibility onto the upconversion nanoparticles in an ultrafast, green and simple way. The pH‐responsive feature of UCTTD‐PC4 remains stable during the blood circulation, while rapidly releases Fe^3+^ in the slightly acidic tumor cells, which results in catalyzing H_2_O_2_ to produce O_2_ and overcoming the tumor hypoxia. Notably, the emission spectrum of the UCTTD perfectly matches the absorption spectrum of the photosensitizer (perylene probe (PC4)) to achieve the enhanced therapeutic effect triggered by a single laser. This study provides a new strategy for the rational design and development of the safe and efficient single near‐infrared laser‐triggered synergistic treatment platform for hypoxic cancer under the guidance of multimodal imaging.

## Graphical abstract

An intelligent dual‐targeted nanosystem is designed based on upconversion nanoparticles coated with the TA/Fe^3+^ coordination film through a one‐step assembly method. This not only can realize acidic tumor microenvironment responsive photosensitizer (perylene probe (PC4)) and Fe^3+^ release, but also simultaneously catalyzes the overexpressed H_2_O_2_ to generate O_2_ for highly efficient single near‐infrared laser‐triggered four‐modal imaging‐guided photothermal therapy/photodynamic therapy synergistic therapy against hypoxic cancer.

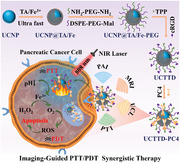

## 1 Introduction

Tumor microenvironment (TME) featuring acidosis, hypoxia, and H_2_O_2_ accumulation, provides an internal environment for the origin and residence of cancer cells.^[^ ^1^ ^]^ The malignant growth, invasion, and metastasis of tumors are inseparable from the characteristics of TME.^[^ ^2^ ^]^ In particular, the hypoxic environment in solid tumors has been proved to be a tricky defect that seriously affects the efficiency of O_2_‐depending photodynamic therapy (PDT), which is a localized treatment method based on the reactive oxygen species (ROS) produced by photosensitizers (PS) in response to light. Because of its negligible invasiveness, high selectivity and minimized side effects, PDT has been increasingly used in tumor treatment.^[^ ^3^ ^]^ With the rapid development of nanomedical technology, the intelligent nanomaterials with TME stimulation response while overcoming tumor hypoxia is considered to be one of the most ideal materials for PDT to achieve precise and efficient cancer therapy.^[^ ^1^, ^4^ ^]^ Therefore, it is urgently desirable for the development of facile strategies that can manufacture such nanosystems with inherent TME‐specific responsivity and achieve satisfactory theranostic results.

The tannic acid (TA), a natural plant polyphenol, is widely used in food and medicine owing to its availability, excellent water solubility, and negligible toxicity.^[^ ^5^ ^]^ At ambient temperature, as an organic ligand, TA can form stable coordination complex with inorganic cross‐linker agents such as Fe^3+^ under neutral pH.^[^ ^6^ ^]^ This method is simple, fast, and requires no special equipment. It only takes a few seconds to deposit TA/Fe^3+^ nanofilms onto different substrates.^[^ ^7^ ^]^ Furthermore, the TA/Fe^3+^ nanofilms are quite stable under neutral conditions, while tend to disintegrate with the decrease of pH,^[^ ^8^ ^]^ which allows them to intelligently expose the payloads in the acidic tumors rather than that during the blood circulation. Besides, it has been reported that some Fe^3+^ doped blocks possessing the catalase‐like activity can catalyze overexpressed H_2_O_2_ (100 µm ^–1^ mm) in the tumors to generate oxygen in situ, alleviate hypoxia and enhance the output of PDT.^[^ ^9^ ^]^ Even more surprisingly, the TA/Fe^3+^ nanofilm can efficiently convert light into heat, thanks to its near‐infrared (NIR) absorption, making the nanofilm a promising therapeutic agent for photothermal therapy (PTT).^[^ ^10^ ^]^ As known, PTT is used for killing cancer cells directly from the heat converted by light at the lesion site.^[^ ^11^ ^]^ Due to minimal invasiveness and great efficiency of PTT in solid tumor elimination, it has attracted much attention among the strategies of cancer treatment^[^ ^12^ ^]^ and becomes a new clinical treatment scheme.^[^ ^13^ ^]^

PTT or PDT has its own unique advantages. However, some researchers gradually realized the insufficient therapeutic efficacy of mono‐phototherapy. To overcome the limitations, some studies have tried PTT/PDT combination therapy to achieve better curative effects.^[^ ^14^ ^]^ While, another obstacle has to be eliminated is that the majority of the synergistic PTT/PDT therapies require two lasers with different wavelengths to activate PTT and PDT separately,^[^ ^15^ ^]^ which increases not only the complexity and the time of the treatment but also the risk of laser exposure, resulting in the limited clinical applications.^[^ ^16^ ^]^ Meanwhile, the issue for inherent defect of the poor penetration depth of the external light at visible or ultraviolet spectral region for PDT has not been well resolved yet. Hence, the development of the effective single NIR laser‐induced synergistic PDT/PTT strategies is highly desirable.^[^ ^17^ ^]^ Up to now, a lot of efforts have been put into the fabrication of multifunctional PSs, hoping to realize the collaborative therapy and solve the problem of laser penetration through a single NIR laser.^[^ ^18^ ^]^ Among these attempts, lanthanide‐doped upconversion nanoparticles (UCNPs) with narrow emission peaks, prominent photostability, and high tissue penetration depth can convert NIR light into visible or ultraviolet emission, which is a nice choice for exciting the PSs to realize PTT/PDT synergistic treatment under a single‐beam NIR laser.^[^ ^19^ ^]^

**Scheme 1:**
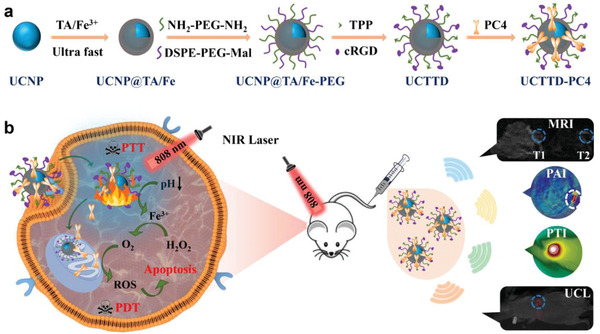
Schematic diagrams of the synthesis and application of UCTTD‐PC4 nanoplatform. a) The synthesis process of UCTTD‐PC4. b) Schematic illustration of the mechanism of tumor‐targeted PTT/PDT for hypoxic pancreatic cancer triggered by TME and guided by multimodal imaging (MRI, PAI, PTI, UCLI).

Inspired by the above background, we prepared a smart dual‐targeted nanosystem with TME stimuli‐responsive and deep tumor penetrability for PTT/PDT cotherapy under the guidance of four‐mode imaging to conquer hypoxia cancers (**Scheme** **1**). In brief, TA/Fe^3+^ nanofilms were coated onto UCNPs via the rapid one‐step assembly to build UCNP@TA/Fe. Then UCNP@TA/Fe nanoparticles were linked to two targeted molecules TPP (4‐carboxybutyl triphenylphosphonium bromide) and cRGD (cyclo (Arg‐Gly‐Asp‐*d*‐Phe‐Cys)), and then loaded with photosensitizer (perylene probe, PC4) to form UCNP@TA/Fe‐TPP‐RGD‐PC4 (UCTTD‐PC4) (Scheme 1a). Through such a simple construction step, the resulted nanoplatform is endowed with many notable merits: 1) the coating of TA/Fe^3+^ nanofilms gives nanoparticles photothermal therapy capacity; 2) UCTTD with dual targeting capability can be precisely delivered to targeted tumor cells and mitochondria with the guidance of RGD and TPP, respectively; 3) UCTTD reduces toxic side effects to normal tissues for its keeping stability under neutral conditions, and selectively releasing PC4 and Fe^3+^ in tumors due to the lower pH stimulation of TME; 4) the contained Fe^3+^ in the nanoassembly can catalyze endogenous H_2_O_2_ into O_2_ to alleviate tumor hypoxia and enhance the therapeutic effect of PDT; 5) the emission spectrum of UCTTD overlaps with the absorption spectrum of PC4, and the PTT/PDT synergistic effect can be activated by the irradiation of a single NIR laser at 808 nm, which simplifies the treatment process, reduces the side effects of extra laser exposure, and improves the therapeutic efficacy by increasing the depth of penetration; 6) UCTTD‐PC4 realizes the four‐modal accurate imaging, including magnetic resonance imaging (MRI), photoacoustic imaging (PAI), photothermal imaging (PTI), and upconversion luminescent imaging (UCLI), and guides the PTT/PDT synergistic cancer treatment (Scheme 1b). All the above results have been proved by the in vivo and in vitro experiments. In short, this work has developed a simple and ingenious method to create a collaborative PTT/PDT nanoplatform that can achieve TME stimulus‐responsive hypoxic cancer treatment under the guidance of four‐modal imaging by a single NIR laser irradiation. It provides a promising choice for a wide range of applications in the further clinical medicine.

## 2 Results and Discussion

### 2.1 Characterization of UCTTD and UCTTD‐PC4

Scheme 1 illustrates the synthetic process of the nanosystem and its working diagram in the diagnosis and treatment of cancer. First, the NaYF_4_:Yb, Tm upconversion nanoparticles (UCNPs) with quite uniform size around 80 nm were synthesized and verified by the high‐resolution transmission electron microscopy (HR‐TEM) (Figure S1a, Supporting Information) and scanning electron microscopy (SEM) (Figure S1b, Supporting Information). The scanning transmission electron microscopy with energy‐dispersive X‐ray spectroscopy (STEM‐EDX) images of the UCNPs show the inner uniform distribution of F, O, Y, Na, Tm, Yb, and the peripheral existence of Nd elements (Figure S1c, Supporting Information). Then, we prepared UCNP@TA/Fe nanoparticles by the one‐step assembly of the TA/Fe^3+^ coordination nanofilms onto UCNPs within 1 min. Figure S2 of the Supporting Information shows that the color of the solution changed from light yellow to black, and the UCNP@TA/Fe nanoparticles became water‐soluble after coating of the nanofilms. Importantly, there is no significant reduction in the intensity of upconversion luminescence (UCL) during the latter modifications (Figure S3a, Supporting Information).

**Figure 1:**
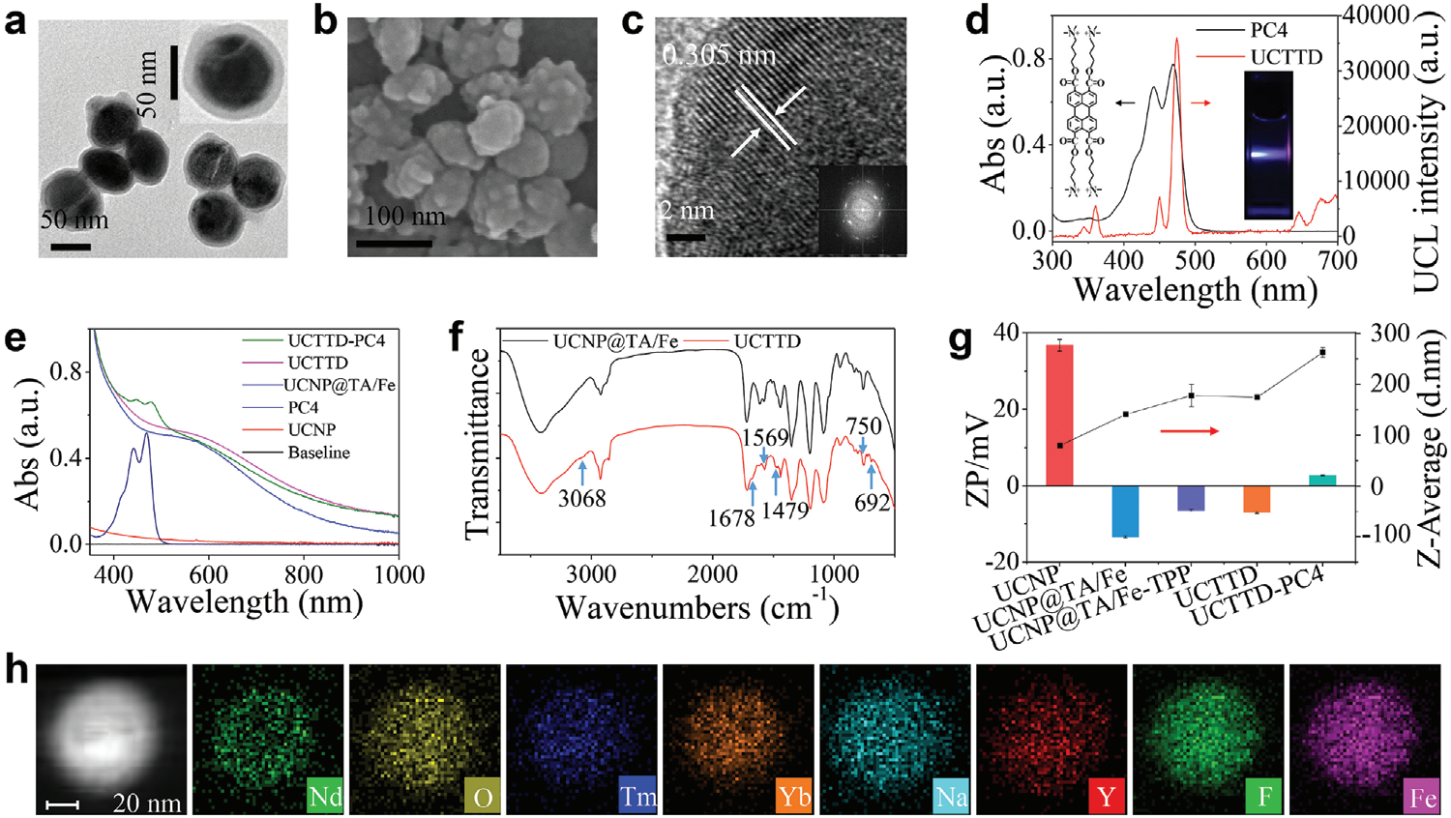
Characterization of the nanoparticles. a) TEM image and b) SEM image of the UCTTD. c) STEM image of UCTTD and the fast Fourier transform (FFT) pattern (inset). d) UV–vis absorption spectrum of PC4 and fluorescence spectrum (*E_x_* = 808 nm) of UCTTD. The inset presents UCL image of UCTTD in vitro. e) UV–vis spectra of UCNPs, PC4, UCNP@TA/Fe, UCTTD, and UCTTD‐PC4. f) FTIR spectra of UCNP@TA/Fe and UCTTD. The peaks at 3068, 1569, 1479, 750, and 692 cm^−1^ correspond to the benzene ring from the TPP, and peak at 1678 cm^−1^ corresponds to the carbonyl group (O=C—OH) from the cRGD. g) Hydrodynamic diameters (line graph) and zeta potentials (histogram) of UCNPs, UCNP@TA/Fe, UCNP@TA/Fe‐TPP, UCTTD, and UCTTD‐PC4. h) High‐angle annular dark field scanning transmission electron microscopy (HAADF‐STEM) and elemental mapping images of an individual UCTTD nanoparticle.

In addition, compared with UCNP@TA/Fe, the Fourier transform infrared (FTIR) spectrum of UCNP@TA/Fe‐TPP‐RGD (UCTTD) (**Figure** **1**f; Figure S3b, Supporting Information) has extra peaks at ≈3068, 1569, 1479, 750, and 692 cm^−1^, corresponding to benzene ring from the TPP, and 1678 cm^−1^ corresponding to the carbonyl group (O=C—OH) from the cRGD, which indicates the successful linking of the target molecules: TPP and RGD peptide. The modification of the UCNPs is also confirmed by the increase in particle size and the change of the surface charge (Figure 1g). SEM and TEM images show that the as‐synthesized UCTTD particles are coated with complex films and have monodisperse morphology with uniform size ≈174 nm (Figure 1a,b). The nanocrystals are also verified by HR‐TEM and shown in Figure 1c. Notably, the STEM‐EDX images (Figure 1h, Figure S1d, Supporting Information) show that UCTTD has an additional Fe element compared to that of UCNPs (Figure S1C, S4, Supporting Information), confirming that TA/Fe complex films are successfully coated on UCNPs. The element distributions are in good agreement with the designed core–shell structure of UCNPs. The cationic perylene probe, PC4 (Figure 1d, inset) is found to be a photosensitizer with excellent chemical and photostability, and efficient ROS generation ability.^[^ ^20^ ^]^ It can be effectively loaded onto UCTTD through electrostatic action, which is verified with the UV–vis spectra (Figure 1e) and the change of surface zeta potential of the UCTTD shifts from −7.0 to 2.7 mV (Figure 1g). The UCTTD‐PC4 possesses the characteristic absorption peaks of both PC4 and UCTTD (Figure 1e), and the strong NIR absorption of UCTTD at ≈808 nm can be used in the further PTT treatment. Figure 1d shows that the emission wavelength of UCL and the maximum absorption of PC4 are both at 470 nm. The perfect spectral overlap is especially important for improving the efficiency of PDT triggered by UCL with irradiation at 808 nm, which also indicates that single wavelength irradiation can achieve both PTT and PDT. The in vitro UCL image of UCTTD (Figure 1d, inset) demonstrates the prominent upconversion luminescent imaging capability. The above characterization results adequately proved that the UCTTD‐PC4 was successfully synthesized.

### 2.2 Photothermal Performance of UCTTD

**Figure 2:**
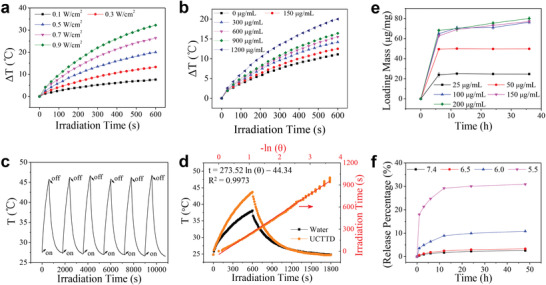
a) Time‐dependent temperature increase of UCTTD solution under NIR laser irradiation with various power intensity (0.1, 0.3, 0.5, 0.7 or 0.9 W cm^−2^) and b) different concentrations of UCTTD (0, 150, 300, 600, 900 or 1200 µg mL^−1^) irradiated identical laser power (0.5 W cm^−2^). c) Photothermal stability of UCTTD solution (1.2 mg mL^−1^) upon 10 min laser irradiation (0.5 W cm^−2^) and 10 min cooling for six on/off cycles. d) Irradiation‐cooling curve of UCTTD solution irradiated with an 808 nm laser at 0.5 W cm^−2^, and the linear fit of time/−ln (*θ*) gained from the cooling process. e) The loading mass curve of PC4 at the concentrations of 25, 50, 100, 150 or 200 µg mL^−1^ to the UCTTD. f) The release behavior of PC4 from the UCTTD in pH 7.4, 6.5, 6.0, and 5.5 PBS measured by the UV–vis spectrometer.

Due to the strong absorption ability of the UCTTD at 808 nm, the NIR irradiation laser was used to excite the nanosystem to generate heat and ROS for PTT and PDT, respectively. As shown in **Figure** **2**a,b, the temperature changes of the UCTTD solutions indicate that the photothermal conversion behavior of the UCTTD is in a laser power density‐ and concentration‐dependent manner. In addition, since the photothermal stability of the PTT agent is another critical factor to ensure efficient PTT therapy, we further measured the temperature variation curves of the UCTTD by repeated exposure to the laser at the intensity of 0.5 W cm^−2^. As shown in Figure 2c, the temperature of UCTTD solution can still reach the initial height after 6 cycles of consecutive laser on/off. Moreover, the photothermal conversion efficiencies (*η*) of UCTTD are as high as 77.86% (Figure 2d). In summary, UCTTD is an excellent PTT reagent with good photothermal performance and photothermal conversion efficiency, which will be eminently suitable for the following application of tumor eradication.

### 2.3 PC4 Loading and Release

The loading mass of PC4 onto UCTTD increased with the increment of PC4 concentration, and reached the maximum value of 80 µg mg^−1^ when the PC4 concentration was 200 µg mL^−1^ (Figure 2e). It is noteworthy that the TA/Fe nanofilm is sensitive to pH.^[^ ^21^ ^]^ Therefore, the release behavior of PC4 in different pH solution was first studied. As shown in Figure 2f, few of the PC4 was released from UCTTD in neutral PBS, which proves that the UCTTD‐PC4 has good stability in physiological environment avoiding the leakage of PC4 before reaching the lesion area. When UCTTD‐PC4 is incubated in PBS solution at pH 6.0 and 5.5 for 48 h, the release efficiency PC4 is up to 10.8% and 30.9%, respectively. The above results indicated that the nanosystem can realize the beneficial pH‐responsive release in the acidic environment of tumor.

### 2.4 Oxygen Generation

**Figure 3:**
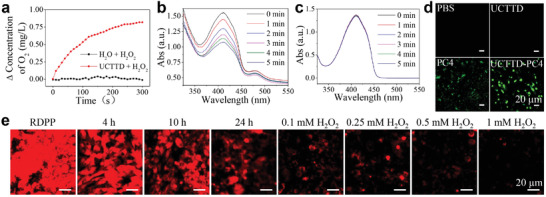
a) Time‐dependent O_2_ generation through catalysis of H_2_O_2_ (0.25 mm) by UCTTD measured by a dissolved oxygen meter. The UV–vis absorption spectra of DPBF solution containing H_2_O_2_ (1 mm) b) with and c) without UCTTD‐PC4 (1000 µg mL^−1^) after irradiation with 808 nm at 0.3 W cm^−2^ for different times. d) Confocal fluorescence images of ROS generation in Capan‐1 cells under different treatments (PBS, UCTTD+L, PC4+L_470_, and UCTTD‐PC4+L; L: 808 nm laser). e) Confocal images of intracellular RDPP fluorescence variation indicating O_2_ content at different time points (0, 4, 10, and 24 h) or at 24 h in the presence of H_2_O_2_ (0.1, 0.25, 0.5 or 1 mm). Scale bar: 20 µm.

The catalase‐like activity of UCTTD was evaluated by real‐time monitoring the dissolved oxygen content. After the addition of UCTTD to H_2_O_2_ (250 µm) solution, a continuous increase in dissolved oxygen concentration was observed in a time‐dependent manner over the following 5 min (**Figure** **3**a), which was ascribed to the presence of Fe^3+^ in UCTTD. As comparison, no O_2_ was generated in the solution without UCTTD. The result confirms that the UCTTD can produce O_2_ efficiently in the presence of H_2_O_2_.

Hypoxic cells in solid tumors are characterized by relative higher concentrations of H_2_O_2_.^[^ ^22^ ^]^ Inspired by this, Fe^3+^ in UCTTD‐PC4 can be used to catalyze H_2_O_2_ to produce O_2_ in situ, thereby enhancing the effect of PDT through self‐supply of O_2_ after the excitation of the PC4. This was also first verified in Capan‐1 cells by the O_2_ probe [Ru(dpp)_3_]Cl_2_ (RDPP),^[^ ^23^ ^]^ whose red fluorescence can be quenched by O_2_ produced in the cells. Obviously, as the incubation time increased to 24 h, the red fluorescence of the cells treated with UCTTD gradually diminished compared with that of cells only treated with RDPP (Figure 3e). Especially, when H_2_O_2_ concentration increased successively, the fluorescence of RDPP was almost completely quenched when the concentration of H_2_O_2_ reached 1 mm. This proves that intracellular O_2_ is produced due to the catalase‐like activity of UCTTD, which will prominently overcome the hypoxia of cancer cells.

### 2.5 UCTTD‐PC4 Amplifies ROS Generation in Mitochondria

The level of ROS production can indirectly reflect the local oxygen content and PDT efficiency. The ^1^O_2_ generation capability of the UCTTD‐PC4 composite was detected under 808 nm irradiation using the 1,3‐diphenyl isobenzofuran (DPBF) as a ^1^O_2_ probe, and the decrease of DPBF absorbance is resulted from the ROS generation. According to Figure 3b, the absorbance of DPBF in the UCTTD‐PC4+H_2_O_2_ group shows noticeable time dependent decrease at 410 nm under the 808 nm laser irradiation within 5 min, indicating that the laser irradiation can effectively induce the rapid ROS generation of UCTTD‐PC4. By contrast, Figure 3c shows that pure DPBF does not exhibit the photodegradation even under the same power of NIR laser irradiation, confirming the photostability of DPBF.

Next, the ROS generation capacity of UCTTD‐PC4 in Capan‐1 cells was measured using a 2′,7′‐dichlorofluorescein diacetate (DCFH‐DA) fluorescent probe (Figure 3d). The rapidly oxidized of nonfluorescent DCFH to DCF by the produced ROS inside the cells emitting green fluorescence can be observed by a confocal laser scanning microscopy. Under 808 nm laser irradiation, Capan‐1 cells in the PBS and the UCTTD group show no green fluorescence. Meanwhile, the ROS generated from PC4 group irradiated by the 470 nm laser can only excite weak fluorescence, which may be attributed to the weak penetration of the 470 nm laser. By comparison, UCTTD‐PC4+L treated cells have the strongest green fluorescence, suggesting that penetration of NIR laser is deeper, and the UCTTD‐PC4 can further enhance PDT by O_2_ generation in tumor cells to produce more cytotoxic ROS.

Changes in mitochondrial membrane potential (MMP), a hallmark of apoptosis, were measured using 5,5,6,6′‐tetrachloro‐1,1′,3,3′‐tetraethylbenzimidazolium cyanoiodine (JC‐1), a mitochondrial depolarization probe that displays polymeric state at high MMP with red fluorescence or monostatic state at low MMP with green fluorescence. As displayed in Figure S5a of the Supporting Information, in comparison with control cells, the red fluorescence in cells treated with PC4+L, UCTTD+L, and UCT+PC4+L decreased distinctly. For the UCTTD+PC4+L group, the red fluorescence completely disappeared accompanied by the appearance of the strong green fluorescence. This suggested that the synergistic effect between PC4 and Fe^3+^ catalyzing center combined with the dual targeting ability together intensify this mitochondrial dysfunction.^[^ ^24^ ^]^ UCTTD enhance the efficacy of PDT through alleviating the hypoxia environment of cancer cells and increasing the laser penetration for PC4 from 470 to 808 nm. In addition, flow cytometry was used to analyze the targeting ability of UCTTD. As shown in Figure S5b,c of the Supporting Information, the delivery efficiencies of UCTTD‐PC4 and UCTT‐PC4 were 73.8% and 49.8%, respectively. This strongly demonstrates the affinity of RGD to its receptor on the surface of Capan‐1 cells and also proves the reliability of UCTTD nanoparticles for cancer targeting treatment.

### 2.6 In Vitro Therapy of UCTTD‐PC4

**Figure 4:**
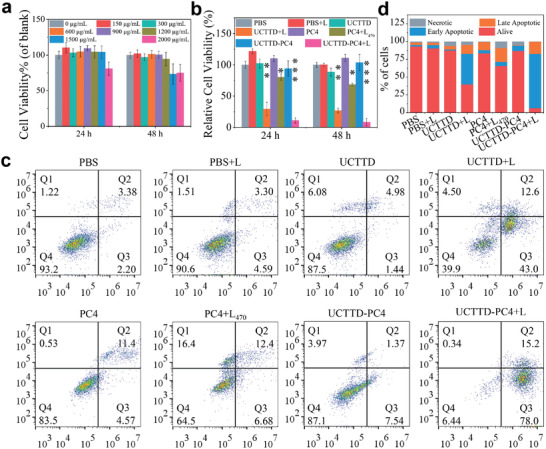
Cell viability and apoptosis measurement after different treatments. a) Relative viabilities of Capan‐1 cells incubated with various concentrations of UCTTD for 24 or 48 h. b) Relative cell viabilities treated with various formulations for 24 or 48 h. c) Representative flow cytometry data of the Annexin V‐FITC and PI costained Capan‐1 cells after incubation with various formulations for 48 h. The number of cells collected in each group was 20 000. Q1, Q2, Q3, and Q4 represent necrotic, late apoptotic, early apoptotic, and living cells, respectively. d) Statistical analysis of cell apoptosis analysis from three independent experiments. Data are presented as means ± SD (standard deviation). L_470_ represents 470 nm laser at power density of 0.08 W cm^−2^, L represents 808 nm laser at power density of 0.3 W cm^−2^ (*n* = 3, *p < 0.05; **p < 0.01; ***p < 0.001).

In order to evaluate the biosafety of UCTTD, we first studied the dose‐dependent cytotoxicity of UCTTD through the cell counting kit‐8 (CCK‐8 kit) under dark conditions. As exhibited in **Figure** **4**a, after incubated with UCTTD with a concentration of less than 1200 µg mL^−1^ for 24 or 48 h, more than 94.5% of the Capan‐1 cells still survived, indicating that UCTTD has extraordinary biocompatibility and biosafety. However, after 808 nm NIR light irradiation just for 10 min, the cells incubated with different formulations for 24 or 48 h showed significantly different cellular viabilities (Figure 4b). Compared with the control group, laser group (PBS+L), materials group (UCTTD, UCTTD‐PC4), and photosensitizer alone group (PC4) had almost no effects on the cell viability, a pronounced reduced to 68.6% and even to 26.8% in cell viabilities were found after treatments with PC4+L_470_ (PDT group) and UCTTD+L (PTT group), respectively. Especially for UCTTD‐PC4+L treatment group, more than 90% of cancer cells were eradicated due to the remarkable effect of PTT/PDT synergistic therapy after irradiation by a single 808 nm NIR laser.

Additionally, Annexin V‐FITC and PI staining experiments were used to further reveal the possible mechanism on the death of the Capan‐1 cells. The flow cytometry analysis results in Figure 4c,d showed that they were almost consistent with the aforementioned cytotoxicity assay results, and the cytotoxicity of UCTTD was almost negligible, while, the PTT/PDT group exhibited the strongest effect in promoting cell apoptosis compared to either of the single treatment (PTT or PDT), and only 6.4% of cells survived at 48 h. This indicates that synergistic therapy with UCTTD may have a good expected effect on in vivo treatment of pancreatic cancer.

### 2.7 Long Circulation and Biodistribution of UCTTD‐PC4

**Figure 5:**
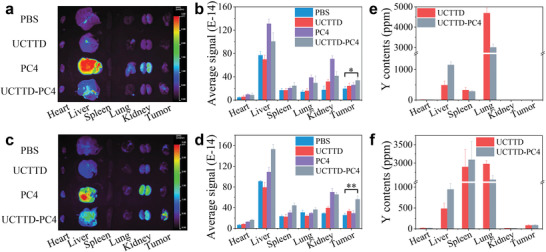
In vivo biodistribution of UCTTD, PC4, and UCTTD‐PC4 in major organs and tumors of Capan‐1 tumor‐bearing BALB/c mice. In vivo biodistribution monitored by the fluorescence of PC4 in major organs and tumors at a,b) 6 h and c,d) 24 h postintravenous injection. Yttrium (Y) contents in major organs and tumors measured by ICP‐MS at e) 6 h and f) 24 h post‐intravenous injection (*n* = 3, *p < 0.05; **p < 0.01).

Long blood circulation and targeted tumor permeability are of great significance for biological applications. Before moving forward to in vivo research on tumor cotherapy, the BALB/c‐nude mice bearing Capan‐1 tumor models were utilized to investigate the in vivo biodistribution behavior of UCTTD, PC4, and UCTTD‐PC4. The content of yttrium (Y), which is the major component of UCNPs, was used as an indicator and measured by ICP/MS to quantify the distribution of UCTTD in main organs and tumors of the mice. The fluorescence of PC4 was used to record the in vivo positions of PC4 and UCTTD‐PC4. As shown in **Figure** **5**a–d, the fluorescence of free PC4 rapidly distributed in the liver at 6 h postinjection, and the fluorescence signal drops rapidly at 24 h due to the metabolic effect of mice. By contrast, after 24 h long circulation in vivo, the fluorescence signal of the UCTTD‐PC4 group was clearly observed at the tumor site due to the targeting property of its RGD peptide and enhanced permeability and retention (EPR) effect, and its intensity was significantly stronger than that of the free PC4 group. The results indicate that UCTTD not only can prolong the blood circulation time of PC4 in vivo, but also possess the capabilities of targeting enrichment as well as long‐term retention in tumors. Moreover, the ICP/MS quantitative analysis data in Figure 5e,f showed that both UCTTD and UCTTD‐PC4 can efficiently accumulate at the tumor site at 24 h postinjection, confirming the above results and demonstrating that UCTTD‐PC4 has a good bioapplication prospect.

### 2.8 In Vitro and In Vivo MRI, PAI, PTI, and Fluorescence Imaging

**Figure 6:**
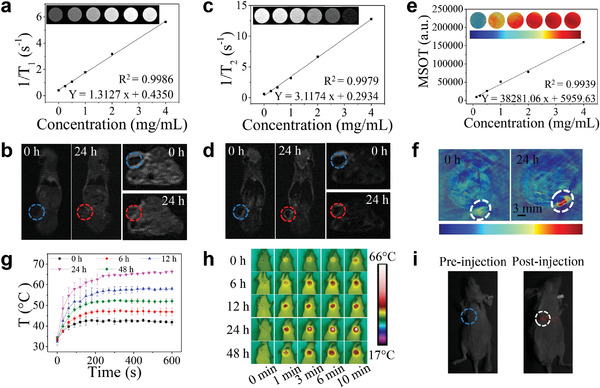
In vitro and in vivo four‐modal imaging. a) *T*_1_‐ and c) *T*_2_‐weighted MR images of UCTTD at different concentrations, and the corresponding longitudinal (r1) and transverse (r2) relaxation rates versus the concentration of UCTTD. b) and d) In vivo MR images of Capan‐1 tumor‐bearing BALB/c mice after the intravenous injection of UCTTD at 0 and 24 h, the blue and red dotted circles represent tumor areas at 0 and 24 h, respectively. e) In vitro PAI images of UCTTD at various concentrations and corresponding plot of the linear relationship between PAI intensity and the concentration. f) PAI images of tumor‐bearing BALB/c mice models after intravenously administered with UCTTD at 0 and 24 h (white dotted circles). h) In vivo infrared thermal images taken at various time points (0, 6, 12, 24, and 48 h) postinjection of UCTTD acquired under 808 nm laser irradiation at power density of 1.2 W cm^−2^ and g) the corresponding tumor temperature curves. i) In vivo UCL images of tumors before (blue dotted circle) and after (white dotted circle) injection of UCTTD.

Accurate imaging is crucial for the early diagnosis, during the treatment and even after the prognosis of cancer.^[^ ^25^ ^]^ In order to reveal the potential of UCTTD as the contrast agent for MRI, the in vitro MRI properties of the UCTTD was first studied by measuring the *T*1‐ and *T*2‐weighted MRI by 3.0 T CX extremely fast nuclear magnetic resonance imaging system. The MR images of UCTTD aqueous solution show an in vitro concentration‐dependent imaging effect. The UCTTD has high longitudinal (*r*1) relativity and transverse (*r*2) relativity, indicating that it is an efficient MRI contrast agent (**Figure** **6**a,c). Furthermore, in vivo MRI tests were performed on the Capan‐1 tumor‐bearing BALB/c mice models after intravenous injection of the UCTTD. As shown in Figure 6b,d, the signal intensities at the tumor sites were significantly enhanced at 24 h postinjection, verifying the high tumor accumulation of UCTTD, which is resulted from the RGD targeting and EPR effect. Next, the PAI potential was further studied by the multispectral optoacoustic tomography imaging system. Prior to in vivo investigation, the in vitro PA signals of artificial models showed a linear dependence on the concentration of UCTTD (*R* ^2^ = 0.9939) (Figure 6e). Thereafter, in vivo PAI of UCTTD was performed on tumor‐bearing BALB/c mice. Figure 6f exhibited distinct PA signals at the tumor site at 24 h postinjection of UCTTD. In addition, we utilized the infrared thermal imaging camera to study the enrichment and photothermal conversion of UCTTD in tumors (Figure 6g,h). The photothermal imaging signal on the tumor site increased significantly with time after intravenous injection of UCTTD and reached the peak at 24 h postinjection. The tumor temperature is ≈60 °C after 808 nm laser irradiation (1.2 W cm^−2^) for 2 min, which is high enough to kill the tumor cells. The tumor accumulation of UCTTD was consistent with the above in vivo imaging results and provided an optimal PTT/PDT therapy time for in vivo therapy. As expected, in vivo UCL imaging signal intensity in tumor was stronger after the injection of UCTTD, indicating the fluorescence imaging ability of the UCTTD in mice (Figure 6i). The above results imply that UCTTD has great application prospects in the field of biological imaging (such as MRI, PAI, UCLI, PTI) and even achieves precise multimodal imaging‐guided therapy.

### 2.9 In Vivo Antitumor Efficiency

**Figure 7:**
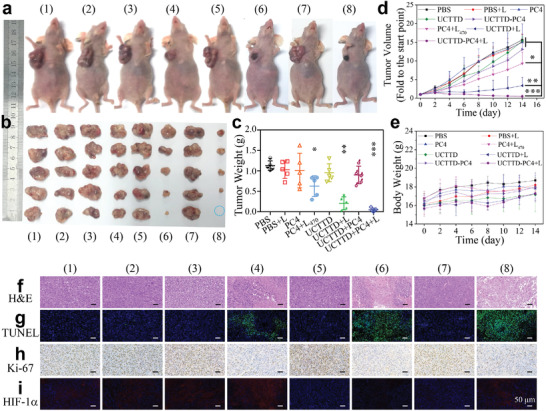
Study on therapeutic effects in vivo. a) Photographs of representative Capan‐1 tumor‐bearing female BALB/c‐nu mice and b) dissected tumors of groups at day 14 after the different treatments. (The blue dotted circle indicates that the tumor has been cured.) c) Tumor weights of mice from different treatment groups on day 14. d) The relative tumor volume and e) mean body weight change curves of mice from different groups during the monitoring period. f) H&E staining images and g) immune‐staining images (TUNEL; h), Ki‐67; i), HIF‐1*α*) of tumor sections from different groups after 14 days of treatment. L_470_ represents 470 nm laser at power density of 0.08 W cm^−2^ for 10 min, L represents 808 nm laser at power density of 1.2 W cm^−2^ for 10 min (*n* = 5, *p < 0.05; **p < 0.01; ***p < 0.001). (1) to (8) represent PBS, PBS+L, PC4, PC4+L_470_, UCTTD, UCTTD+L, UCTTD‐PC4, and UCTTD‐PC4+L, respectively. All scale bars are 50 µm.

Inspired by the remarkable lethality of cancer cells in vitro and the eminent multimodal imaging capabilities, the in vivo synergistic anticancer performances were further confirmed on the Capan‐1 tumor‐bearing female BALB/c‐nude mice models. After the xenograft Capan‐1 tumor volume reached ≈100 mm^3^, the tumor‐bearing mice were randomly divided into eight groups (*n =* 5) for different treatments. Then, the control groups were treated with PBS, PBS+L, PC4, UCTTD or UCTTD‐PC4 and treatment groups were treated with PC4+L_470_ (PDT), UCTTD+L (PTT) or UCTTD‐PC4+L (PDT/PTT). After 24 h of the intravenous injection, the mice were exposed to laser irradiation at 808 nm (1.2 W cm^−2^, 10 min) or 470 nm (0.08 W cm^−2^, 10 min). The tumor volumes and body weights were then recorded every other day for the following 14 days. The photographs of the representative mice and the excised tumors after different treatments at the 14th day (**Figure** **7**a,b), as well as the weight of the tumors (Figure 7c) visually revealed the excellent therapeutic effect of UCTTD‐PC4+L. Figure 7d showed that tumors in the control groups continued to grow rapidly, suggesting that only laser irradiation, photosensitizer alone or nanomaterial alone had no inhibitory effects on tumor growth. Comparatively, the growth rate of tumors in the PC4+L_470_ group was slightly slow due to the PDT effect. Moreover, the PTT efficacy in the UCTTD+L group was better than that of the PDT group and displayed an obviously inhibitory effect on tumor growth. Remarkably, the tumors in PDT/PTT synergetic therapy group were significantly inhibited, the volumes were much smaller after two weeks of therapeutic course with the tumor inhibition rate of 97%, confirming the considerable antitumor effect of the PDT/PTT synergistic therapy.

In order to further determine the antitumor efficacy, we performed pathological evaluation on tumor tissue sections. The hematoxylin and eosin (H&E) staining showed that most tumor cells were severely damaged in the PTT/PDT combination therapy group, followed by PTT group and PDT group (Figure 7f). Moreover, the terminal deoxynucleotidyl transferase‐mediated dUTP‐biotin nick end labeling (TUNEL) stained tumor slice of the PTT/PDT group showed the highest level of apoptosis (Figure 7g). The proliferation of tumors was further evaluated by immunostaining with proliferation marker Ki‐67, which was consistent with the aforementioned results. The proliferation ability of tumor cells in the PTT/PDT group was significantly inhibited compared to other groups (Figure 7h). Besides, the hypoxic tumor microenvironment is known to induce the expression of hypoxia‐inducible factor‐1*α* (HIF‐1*α*).^[^ ^26^ ^]^ HIF‐1*α* immunohistochemistry of tumor sections was used to verify the ability of UCTTD for alleviating tumor hypoxia (Figure 7i). Compared with the blank group or the PDT group that consumes O_2_, a distinct blue fluorescence was found in UCTTD group and UCTTD‐PC4 group. This indicates that UCTTD has catalase‐like activity and can catalyze the decomposition of H_2_O_2_ into O_2_ in situ to overcome tumor hypoxia. The results reveal that UCTTD‐PC4 nanosystem targeting the tumor sites provides an effective method to combat the weak laser penetration and hypoxia inhibited PDT during the in vivo synergistic antitumor process, and markedly inhibit tumor growth without obvious damage to normal tissues.

Moreover, the biosafety of the nanoparticles was further assessed. We found that body weight in each group increased steadily throughout the treatment evaluation period (Figure 7e), certifying the negligible systemic toxicity of these treatments in mice. Besides, the H&E stained sections showed that there were no obvious pathological changes in the main organs (heart, liver, spleen, lung, and kidney) of the mice after treatments for two weeks (Figure S6, Supporting Information). The results once again confirmed the biocompatibility of the nanoparticles in each group during the antitumor therapy. Additionally, the serum biochemical parameters including creatine phosphokinase, alanine aminotransferase, aspartate transaminase, and blood urea nitrogen had almost no abnormal changes compared with the PBS groups, implying that the function of the heart, liver, and kidney of mice in each group was not significantly affected after treatment (Figure S7, Supporting Information). In summary, these results fully suggest that the prepared UCTTD‐PC4 has remarkable antitumor effects and favorable biosafety in mice, and is expected to be used in the synergistic PTT/PDT of cancer therapy under the guidance of multimodal imaging.

## 3 Conclusion

In conclusion, we have successfully prepared an intelligent anticancer theranostic nanosystem based on UCNPs coated with coordination complexes formed by Fe^3+^ and TA through the one‐step assembly method at room temperature. The UCTTD‐PC4 can be specifically and efficiently delivered into tumor cells and mitochondria due to the dual targeting capabilities. Upon exposure to NIR irradiation, it not only can achieve the photothermal conversion for PTT, but also motivate PC4 for PDT, and finally realize the synergistic tumor therapy triggered by a single laser. Significantly, introducing Fe^3+^ can catalyze H_2_O_2_ to generate O_2_, and complexes with TA to form a nanofilm with obvious pH sensitivity that can respond to the slightly acidic environment of the tumor, resulting in releasing PC4 effectively at the tumor site and enhancing therapeutic efficiency. The in vivo results confirm that the nanosystem has predominant four‐modal imaging ability, superior synergistic PTT/PDT antitumor effect under a single laser and negligible systemic toxicity. Overall, the facile fabrication and TME‐responsive multifunctional nanoplatform opens a new avenue for designing photonanomedicine in anticancer and for extensive biomedical applications in versatile research fields.

## Conflict of Interest

The authors declare no conflict of interest.

## Supporting information

Supporting Information

## Data Availability

The data that support the findings of this study are available from the corresponding author upon reasonable request.
