# Supplementary material for: Tumor Microenvironment Stimuli‐Responsive Single‐NIR‐Laser Activated Synergistic Phototherapy for Hypoxic Cancer by Perylene Functionalized Dual‐Targeted Upconversion Nanoparticles
Source: Adv Sci (Weinh). 2022 Aug 28;9(30):2203292. doi: 10.1002/advs.202203292 (PMC9596832; doi:10.1002/advs.202203292)
Supplement: Supplementary file 1 — Supporting Information [file ADVS-9-2203292-s001.pdf]

ADVANCED SCIENCE
Open Access

Supporting Information

for *Adv. Sci.*, DOI 10.1002/advs.202203292

Tumor Microenvironment Stimuli-Responsive Single-NIR-Laser Activated Synergistic Phototherapy for Hypoxic Cancer by Perylene Functionalized Dual-Targeted Upconversion Nanoparticles

Xiuna Jia, Deming Liu, Cong Yu, Niu Niu, Dan Li, Jin Wang* and Erkang Wang*

Supporting Information

Tumor Microenvironment Stimuli-Responsive Single-NIR-Laser Activated Synergistic Phototherapy for Hypoxic Cancer by Perylene Functionalized Dual-Targeted Upconversion Nanoparticles

*Xiuna Jia [1], Deming Liu [2], Cong Yu [1], Niu Niu [1], Dan Li [1 *], Jin Wang [3 *], and Erkang Wang [1, 4]*

[1] State Key Laboratory of Electroanalytical Chemistry, Changchun Institute of Applied Chemistry, Chinese Academy of Sciences, Changchun, Jilin, 130022, P. R. China

[2] State Key Laboratory of Luminescence and Applications, Changchun Institute of Optics, Fine Mechanics and Physics, Chinese Academy of Sciences, Changchun, Jilin, 130033, P. R. China

[3] Department of Chemistry and Physics, State University of New York at Stony Brook NY 11794-3400, USA

[4] College of Chemistry, Jilin University, Changchun, Jilin, 130012, P. R. China

Experimental Section

Chemicals and Materials

Rare earth chlorides ($LnCl_3$, Ln: Y, Yb, Tm, Nd) were supplied by Sigma-Aldrich (St. Louis, MO, USA). Oleic acid (OA), octadecene (ODE), (4-carboxybutyl) triphenylphosphonium bromide (TPP) and tannic acid were purchased from Aladdin Industrial Inc. (Shanghai, China). Ammonium fluoride (NH_4F), methyl alcohol (CH_3OH), sodium hydroxide (NaOH), methylene chloride (CH_2Cl_2), ferric chloride ($FeCl_3$) and cyclohexane were obtained from Sinopharm Chemical Reagent Co., Ltd. (Shanghai, China). N-(3-dimethylaminopropyl)-N-ethylcarbodiimide hydrochloride (EDC), dimethyl sulfoxide (DMSO), 1,3-Diphenylisobenzofuran (DPBF), N-Hydroxysuccinimide (NHS) NH_2-PEG_{2000}-NH_2 and trypsin were purchased from Sigma-Aldrich (USA). Nitrosonium tetrafluoroborate ($NOBF_4$) was purchased from Alfa Aesar Chemical Co. Ltd (Tianjing, China). cRGDfc [Cyclo(Arg-Gly-Asp-D-Phe-Cys)] was synthesized and purified by Sangon Biotech Co., Ltd. (Shanghai, China). A cell counting kit-8 (CCK-8) kit was purchased from Dojindo (Japan). ROS fluorescence probe (2,7-dichlorodihydrofluorescein diacetate, DCFH-DA) and enhanced mitochondrial membrane potential assay kit (JC-1) were purchased from Beyotime Biotechnology (Shanghai, China). Annexin V-FITC/PI kit and corning matrigel basement membrane matrix were obtained from Becton Dickinson and Company (USA). DSPE-PEG_{2000} and DSPE-PEG_{2000}-Maleimide were obtained from Nanocs Inc. (USA). Iscove's Modified Dulbecco's Medium (IMDM) and fetal bovine serum (FBS) were supplied from Gibco (Grand Island, NY, USA). The Capan-1 cells were obtained from the Kunming Institute of Zoology, Chinese Academy of Sciences (China). The photosensitizer perylene probe (PC4) came from the research group of Prof. Yu Cong, Changchun Institute of Applied Chemistry,

Chinese Academy of Sciences (China). All chemicals were used as received without any further purification.

Synthesis of $NaYF_4$: Yb, Tm Upconversion Nanoparticles (UCNPs)

$NaYF_4$: Yb, Tm upconversion nanoparticles were synthesized *via* a previously procedure with some modifications.[1] In a typical process, 1 mmol rare-earth chlorides (0.795 mmol Y^{3+}, 0.2 mmol Yb^{3+} and 0.005 mmol Tm^{3+}) were added to a 100 mL three-necked flask containing 6 mL oleic acid (OA) and 15 mL 1-octadecene (ODE). The solution under an argon atmosphere with vigorous stirring for 10 min, and then was heated to 170°C for 60 min before cooled down to room temperature. Subsequently, 10 mL methanol solution containing NaOH (0.1 g) and NH_4F (0.148 g) was added slowly and stirred for another 30 minutes. Thereafter, the solution was degassed at 110°C for 30 min to remove the methanol and water. Then the solution was heated to 150°C and maintained for another 10 min. Then, the solution was heated to 300°C and keep for 90 min under an argon atmosphere. The nanoparticles were precipitated with ethanol, washed with cyclohexane/ethanol (1:2, *v/v*) three times, and then re-dispersed in cyclohexane.

In order to enable the nanoparticles to absorb 808 nm laser, we coated a $NaYF_4$: 20% Nd shell onto the above nanoparticles. Generally, 0.06 mmol $NdCl_3$, 0.24 mmol YCl_3, were added into a 100 mL three neck round bottom flask containing 5 mL OA and 6 mL ODE. The solution was heated to 110°C and maintained for 10 min under an argon atmosphere with stirring. Then it was heated to 170°C and kept for 60 min before cooling down. When the temperature of the solution was lowered to 60°C, 20 mg of $NaYF_4$: 0.05% Tm^{3+}, 20% Yb^{3+} nanoparticles was added, and heated to 150°C, the subsequent process is the same as the above operation to obtain the final upconversion nanoparticles.

Ligand-Exchange Reactions of UCNPs

Briefly, 5 mL of dichloromethane solution of $NOBF_4$ (0.01 M) was added to 5 mL of UCNPs dispersion in hexane (5 mg/mL) at room temperature. The resulting mixture was shaken gently and sonicated in an ice-water bath until the precipitation of UCNPs was observed. After being washed 3 times with absolute ethanol, the precipitated UCNPs were re-dispersed in water.

Preparation of UCNP@TA/Fe

The nanofilm was conformally coated according to previous reports.[2] The 5 mg UCNPs were added to 1 mL of natural polyphenol tannic acid (TA) solution (3.2 mg/mL), followed by 10 min of ultrasound. Then, 0.5 mL $FeCl_3$ solution (1.6 mg/mL) was added under vigorous magnetic stirring or fast stirring for 1 min at ambient temperature, and the mixture turned black immediately. Afterwards, they were centrifuged quickly and rinsed twice with 75% ethanol and once with deionized water to obtain UCNP@TA/Fe.

Preparation of UCNP@TA/Fe-TPP-cRGD (UCTTD)

In order to give the UCNP@TA/Fe targeting ability, mitochondrial targeting molecule TPP and RGD peptides that recognize the overexpressed $\alpha v\beta_3$ integrin receptor on the surface of cancer cells were linked by amination reaction and Michael addition reaction, respectively, to prepare the UCNP@TA/Fe-TPP-RGD (UCTTD). 1 mg of PEG (DSPE-PEG_{2000}: NH_2-PEG_{2000}-NH_2: DSPE-PEG_{2000}-Maleimide = 90 : 5 : 5, wt ratio) was firstly added into 1 mL of UCNP@TA/Fe (5 mg/mL) in 18.2 Ω water (pH = 7.2) (DSPE-PEG_{2000} and DSPE-PEG_{2000}-Maleimide were obtained from Nanocs Inc., NH_2-PEG_{2000}-NH_2 was purchased from Sigma-Aldrich). The mixture was rotated and shaken for 12 h, and then washed twice with deionized water. At the same time, TPP (0.2 mmol), EDC (0.5 mmol) and NHS (1 mmol) were dissolved in DMSO (100 µL).

After stirring in the dark for 2 h, the activated TPP was added to 1 mL of PEG-modified UCNP@TA/Fe mentioned above. After overnight reaction at room temperature, the UCNP@TA/Fe-TPP was collected by centrifugation and washed with deionized water for 3 times. In order to modify the cRGD targeting protein, UCNP@TA/Fe-TPP was added to the cRGD solution (0.05 mg/mL), rotated at 4°C for 12 h, and then washed with deionized water for 3 times to obtain the UCNP@TA/Fe-TPP-cRGD (UCTTD).

Loading and Release Experiment of the Photosensitizer

To investigate the photosensitizer (PC4) loading and release behavior, 1 mg UCTTD was added to 1 mL of PC4 solution with different concentrations (25, 50, 100, 150, or 200 μg/mL) and constantly shaken in the dark for 36 h at room temperature. The concentration of PC4 (C_x) in the supernatant was measured *via* UV-vis absorption at 6, 12, 24 and 36 h and calculated by the equation: Loading rate = $(C_0-C_x)/C_0 \times 100\%$, where C_0 is the initial concentration of PC4. For the release behavior of PC4, UCTTD-PC4 was placed in PBS solution with pH of 5.0, 6.5 and 7.4, respectively, and the concentration of PC4 (C_x) released in the supernatant was measured at 1, 3, 6, 12, 24 and 48 h (Release rate = $C_x/C_0 \times 100\%$, where C_0 is the initial amount of PC4 has been loaded on UCTTD).

Characterization of Nanomaterials

The HR-TEM images were recorded by a high resolution transmission electron microscope operating at 200 kV (JEM-2100F, Japan). HAADF-STEM images, elemental mappings and energy dispersion X-ray spectra were obtained by high resolution electron microscopy. The surface morphologies of nanocarriers were characterized by scanning electronic microscopy (SEM, XL30 ESEM, Japan). Fourier Transform Infrared (FT-IR) spectra (VERTEX70 FT-IR Spectrophotometer, Bruker

Optics, Germany) was employed to confirm the connection of the targeted molecules. The absorption spectra were measured with a Cary500 Scan UV-vis scanning spectrophotometer (Varian, USA). The fluorescence spectra were carried out on a FLWOROMAX-4 fluorescence spectrometer (PL) (HORIBA). Hydrodynamic diameter and zeta potential were acquired by a Malvern Zetasizer Nano ZS (UK).

Photothermal Performance and Photothermal Stability

One milliliter of the UCTTD solution with different concentrations (0, 150, 300, 600, 900, 1200 µg/mL) were irradiated with an 808 nm laser (LOS2-BLD-0808-005W-C/P, Hi-Tech Optoelectronics Co., Ltd, China) at a power density of 0.3 W/cm^2 for 10 min. Meanwhile, the UCTTD solution was irradiated by different laser power (0.1, 0.3, 0.5, 0.7, and 0.9 W/cm^2). Moreover, to investigate the photothermal stability, the UCTTD solution was irradiated six cycles of consecutive laser on/off under similar conditions. The thermocouple probe linked to a digital thermometer (TES K-Type Thermometer 1319A, Taiwan) and an infrared thermal camera (FLIR C2, USA) recorded the real time temperature and infrared thermal images every 30 s. The photothermal conversion efficiency (η) was calculated by the previous method.[3]

Oxygen Production *in Vivo* and *in Vitro*

The extracellular generation of O_2 was measured by YSI5000 dissolved oxygen-BOD tester (USA). Briefly, the UCTTD nanoparticles was added in 30 mL of H_2O_2 solution (1 mM), and the mixture was placed in a 50 mL centrifuge tube. Then, the dissolved oxygen meter probe was used to detect the change of oxygen concentration under constant stirring, and 1 mM H_2O_2 aqueous solution was used as a negative control. Before starting the experiments, all solutions were purged with nitrogen to remove the dissolved oxygen.

The intracellular generation of O_2 was monitored by RDPP reagent and confocal laser

scanning microscope (CLSM, Nikon, Japan) imaging technology. The Capan-1 cells were incubated with 10 μM RDPP for 4 h and then incubated with different concentrations of UCTTD nanoparticles for 4, 10 or 24 h. The cells at 24 h were added with different concentrations of H_2O_2 (0.1, 0.25, 0.5, or 1 mM), and then washed with PBS for three times. Cell only incubated with RDPP (10 μM) was used as the positive control. Finally, confocal fluorescence imaging of RDPP was observed at 488 nm exciation.

Extracellular ROS Detection

ROS production was confirmed through the absorption of chemical probe DPBF at 410 nm by UV-vis spectrophotometer. The 0.5 mL ethanol solution of DPBF (40 μg/mL) was added to 0.5 mL of the UCTTD-PC4 suspension (1000 μg/mL) in 1 mM H_2O_2 aqueous solution. Then the mixture was transferred into a cuvette, and was irradiated with an 808 nm laser (0.3 W/cm^2) for 5 min in the darkness, during which the absorption intensity of DPBF was recorded once per minute. DPBF (20 μg/mL) without UCTTD-PC4 after irradiation was served as the control group.

Intracellular ROS Detection

The intracellular generation of ROS was studied with DCFH-DA Kit by CLSM imaging. The Capan-1 cells were seeded into 96-well plates at the density of 1×10^4 per well. After 12 h of incubation, cells were treated with PBS, UCTTD, PC4 or UCTTD-PC4 for 12 h before washed with PBS, then, the ROS sensor (0.1 μM DCFH-DA) was added to each well. The cells were then incubated for another 20 min before irradiated with an 808 nm laser (0.3 W/cm^2) or 470 nm laser (0.08 W/cm^2) for 10 min. Finally, the intracellular distribution of DCF fluorescence, which represents ROS was recorded *via* CLSM after washing with PBS.

Mitochondrial Membrane Potential Detection

A decrease of the mitochondrial membrane potential is a landmark event of apoptosis. It was measured by the mitochondrial specific fluorescent cationic dye 5,5′,6,6′-tetrachloro-1,1′,3,3′-tetraethylbenzimidazolylcarbocy anine iodide (JC-1). The transition of JC-1 from red fluorescence to green fluorescence indicates the occurrence of cell apoptosis. The fluorescence changes of Capan-1 cells were captured by CLSM. Briefly, the Capan-1 cells were seeded into 96-well plates at the density of 1×10^4 / well overnight, and then co-incubated with PC4, UCTTD, UCNP@TA/Fe-PC4 (UCT-PC4) or UCTTD-PC4, respectively, for 8 h. The laser irradiation was carried out with an 808 nm laser (0.3 W/cm^2) or a 470 nm laser (0.08 W/cm^2) for 10 min. Subsequently, the cells were stained with freshly prepared JC-1 (2.5 μg/mL) at 37°C for 20 min. Finally, the cells were washed with cold JC-1 buffer for 3 times and observed by CLSM to confirm targeted mitochondrial damage. Under the excitation with 488 nm laser, the green light of JC-1 monomers were detected at 529 nm, and the red light of JC-1 aggregates were detected at 590 nm.

Targeted Delivery

The cultured cells were divided into two groups and co-incubated with UCNP@TA/Fe-TPP-PC4 (UCTT-PC4) and UCTTD-PC4 with cRGD-targeted protein for 6 h respectively. After digestion, the cells were quantitatively analyzed by flow cytometry (FCM).

Cytotoxicity Assay

Cell activity and toxicity tests were evaluated *via* CCK-8 kit. The Capan-1 cells were incubated overnight, and then different concentrations of UCTTD (0, 150, 300, 600, 900, 1200, 1500, 2000 μg/mL) were added to text the toxicity of the UCTTD. Another, different formulations (PBS, UCTTD, PC4, UCTTD-PC4) were added and incubated for 6 h, followed by the irradiation of the 808 nm laser (0.3 W/cm^2) or 470 nm laser

(0.08 W/cm^2) for 10 min. After continuing to incubate for 24 h and 48 h, the cells were washed with PBS, and then added 100 μL of fresh culture medium (containing 10 μL of CCK-8 reagent). Finally, the microplate reader (Spark Control^{TM} Tecan, USA) recorded the absorbance at 450 nm. The experiment was repeated three times for each group.

Cell Apoptosis Assay

The Capan-1 cells were seeded in 48-well plates and incubated overnight, and then the cells were treated with PBS, UCTTD, PC4, or UCTTD-PC4 respectively for 6 h, followed by the irradiation of the 808 nm laser (0.3 W/cm^2) or 470 nm laser (0.08 W/cm^2) for 10 min. After continuing to incubate for another 42 h, the cells were collected and washed with PBS. Finally, the apoptosis detection kit was used to evaluate cell apoptosis. The fluorescence of 20, 000 cells were detected using a flow cytometer (BD AccuriTM C6, USA). Non-treated cells were used as the control.

Animal Tumor Model

The tumor models were established on female BALB/c nude mice of 5-6 weeks old (Beijing Vital River Laboratory Animal Technology Co., Ltd., Beijing, China), which were injected with 1 × 10^7 Capan-1 cells in 50 μL of serum-free DMEM medium supplemented with 50 μL of corning matrigel basement membrane matrix into their armpit for cancer treatment study or in their backs for tumor imaging research. All animal experimental procedures were compliant with the guidelines for ethical review of animal welfare (GB/T 35892-2018) and the regulations of the Institutional Animal Care and Use Committee (IACUC) of Changchun WISH Technology Service Co., Ltd.

Biodistribution Assay

The tumor-bearing mice were randomly divided into 4 groups (n = 3 per group) after

the tumor volume reached approximately 200-300 mm^3 to study the biodistribution behavior, the mice were intravenously injected with PBS, PC4, UCTTD or UCTTD-PC4 (5 mg/mL), respectively. Afterward, the main organs such as heart, liver, spleen, lung, kidney, as well as tumors were harvested at 6 and 24 h post-injection respectively. The fluorescence intensity was imaged by Davinch Invivo HR imaging system (Davinch K, Korea). Subsequently, the tumors and other healthy organs were lyophilized, weighed and dissolved for ICP-MS (X Series 2, Thermo Scientific, USA) analysis of Y content.

Photoacoustic Imaging and Photothermal Imaging

The photoacoustic imaging (PAI) capability of the UCTTD was verified by the multispectral optoacoustic tomography imaging system (MSOT inVision 128, iThermedical, Germany). Different concentrations of UCTTD (0.125, 0.25, 0.5, 1, 2, 4 mg/mL) were injected into the prosthesis for PAI. For *in vivo* PAI, when the tumor volume of Capan-1 tumor-bearing nude mice reached about 150 mm^3, 200 µL of UCTTD were injected intravenously at different time points. Then the mice were anesthetized with 2% isoflurane in oxygen and covered with ultrasonic coupling gel. Finally, the results of PA images were recorded by MOST system.

For *in vivo* photothermal imaging, when the tumor-bearing mice were injected intravenously with 200 µL UCTTD (5 mg/mL) at different time points, the mice were anesthetized by intraperitoneal injection of 2% sodium pentobarbital (50 mg/kg), followed by irradiation of 808 nm laser (1.2 W/cm^2). The infrared thermal imaging camera was employed to monitor the tumor temperature and collect photothermal images.

***In Vitro* and *in Vivo* Magnetic Resonance Imaging (MRI)**

In order to measure the relaxation rate of UCTTD, the sample was diluted to 0, 0.5, 1,

2, or 4 mg/mL for *in vitro* magnetic resonance imaging (MRI). For *in vivo* MRI imaging, when the tumor volume of nude mice reached 200-300 mm^3, 200 μL UCTTD (5 mg/mL) was injected intravenously into the mice. At 0 h and 24 h, the mice were anesthetized, followed by 3.0 T CX extremely fast nuclear magnetic resonance (Philips Ingenia) imaging system collects T1- and T2- weighted imaging pictures.

Upconversion Luminescence Imaging (UCL)

Upconversion luminescence imaging was achieved by Davinch Invivo HR imaging system equipped with 808 nm laser. Briefly, when the tumor volume of nude mice reached 100 mm^3, the mice were anesthetized and 50 μL UCTTD was injected intratumoral. Upconversion luminescence signals were recorded in the green channel.

Photothermal and Photodynamic Synergistic Therapy

When the tumor volume of female BALB/c nude mice reached 100 mm^3, they were randomly divided into 8 groups (*n* = 5 for each group): PBS, PBS+L, PC4, PC4+L_{470}, UCTTD (5 mg/mL), UCTTD+L, UCTTD-PC4, UCTTD-PC4+L. The injection dose was 200 μL. Laser irradiation was administered at 24 h post intravenous injection. L represent 808 nm laser (1.2 W/cm^2), L_{470} represent 470 nm laser (0.08 W/cm^2). The tumor volume was examined and calculated as length × $(width)^2$ × 0.5 with a vernier calliper every two days during the treatment period.

The mice were euthanized at 2 weeks after treatments and then the blood was collected for biochemistry analysis. The tumors and organs (liver, heart, spleen, lung, and kidney) in each group were isolated from the mice, and then fixed with 4% neutral buffered formalin and embedded in paraffin for hematoxylin and eosin (H&E) staining, terminal deoxynucleotidyl transferase dUTP nick end labeling (TUNEL) staining, Ki-67 and HIF-1α labeling. Finally, they were examined by the inverted

fluorescence microscope (NIKON, Japan).

Statistical Analysis

All data were presented as mean ± standard deviation (SD) of triplicates for cell experiments or quintuplicates for animal experiments unless otherwise indicated. The differences in experimental data were compared by the One-way ANOVA statistical analysis by the SPSS software (IBM SPSS Statistics, USA). The level of statistical significance was settled at $*p < 0.05$. $**p < 0.01$; $***p < 0.001$.

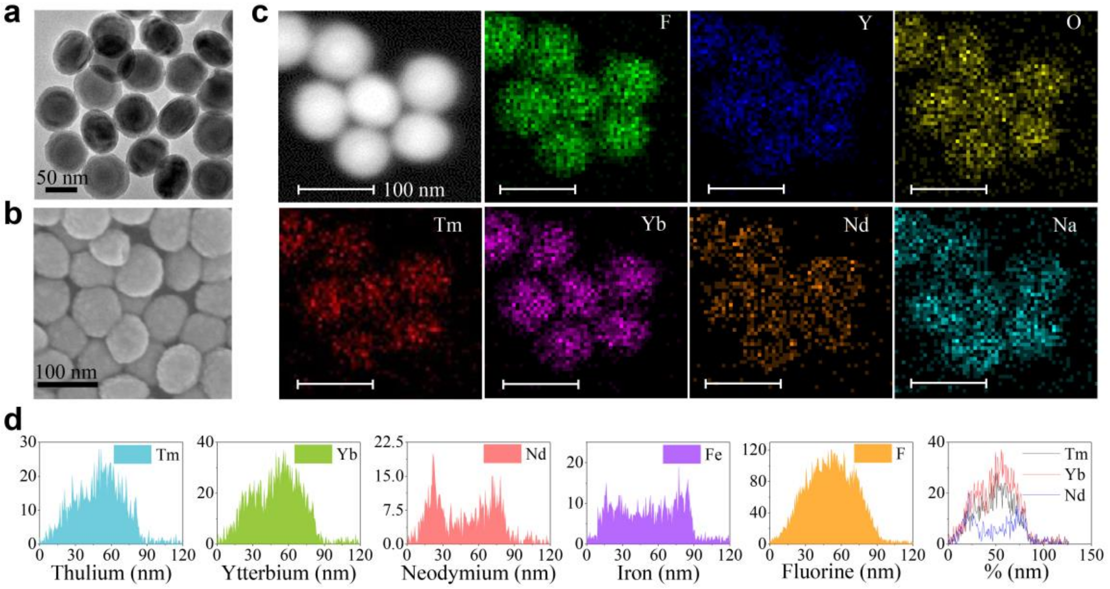

Figure S1. (a) TEM image and (b) SEM image of the UCNPs. (c) HAADF-STEM and the corresponding elemental mapping images of UCNPs. Scale bars are 100 nm. (d) Linear element distribution on one UCTTD nanoparticle.

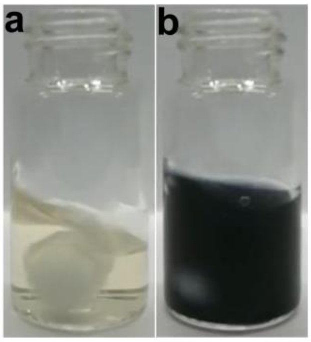

Figure S2. Images of UCNP@TA/Fe nanomaterials before and after coating TA/Fe nanofilms. (a) Image of UCNPs in TA solution. (b) Image of UCNP@TA/Fe solution.

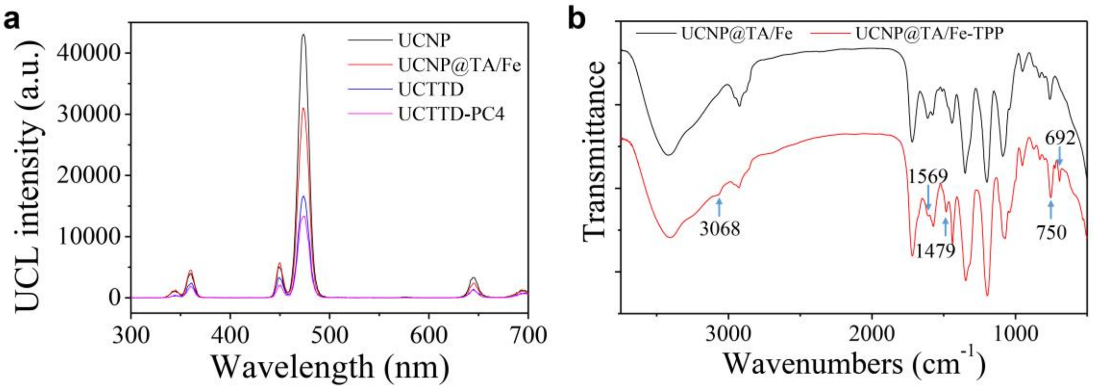

Figure S3. Characterization of the nanoparticles. (a) Fluorescence spectra of UCNPs, UCNP@TA/Fe, UCTTD and UCTTD-PC4 (Ex = 808 nm). (b) FTIR spectra of UCNP@TA/Fe and UCNP@TA/Fe-TPP. The peaks at 3068 cm^{-1}, 1569 cm^{-1}, 1479 cm^{-1}, 750 cm^{-1} and 692 cm^{-1} correspond to the benzene ring from TPP.

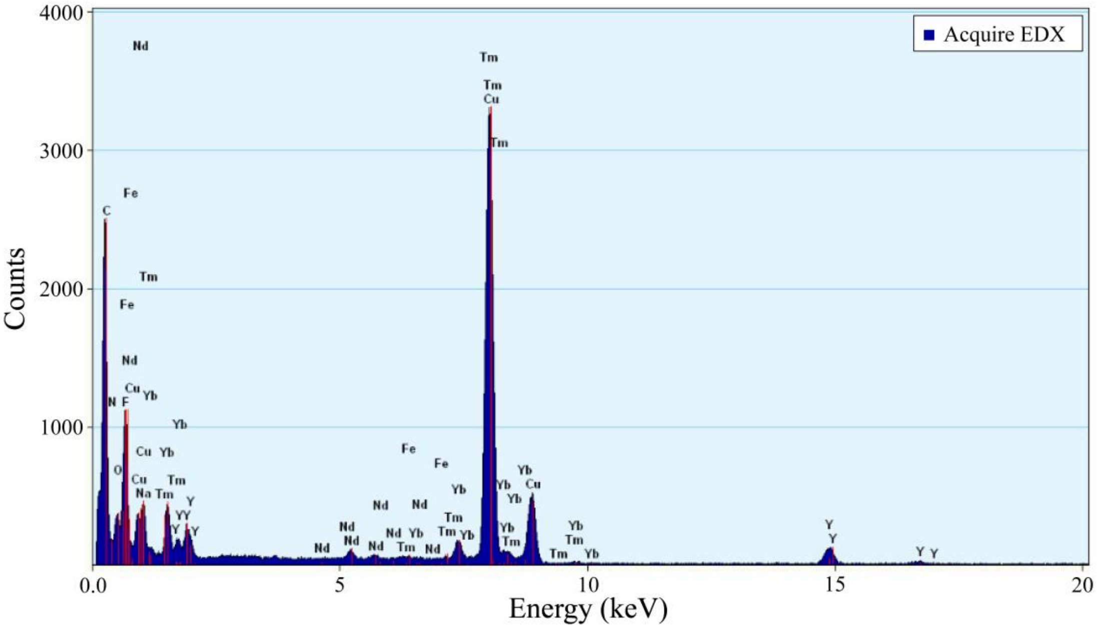

Figure S4. The energy-dispersive X-ray spectroscopy (EDX) of UCTTD.

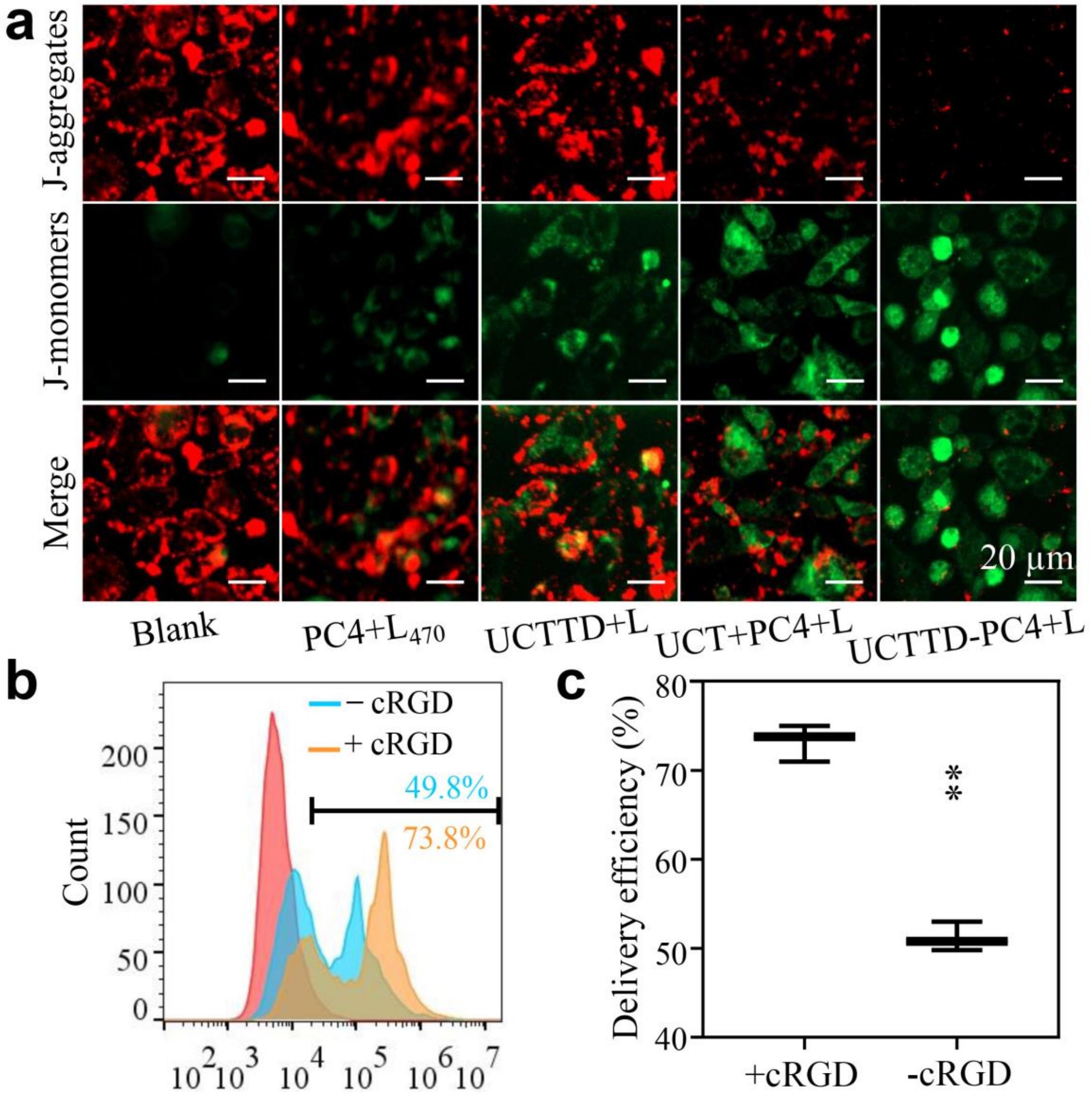

Figure S5. Study on mitochondrial potential and RGD targeting ability. (a) Confocal fluorescence images of mitochondrial membrane potential changes in cells incubation with JC-1 after treatments with PBS, PC4+L_{470}, UCTTD+L, UCT-PC4+L or UCTTD-PC4+L for 8 h. Scale bar: 20 µm. (b) Quantitative determination of intracellular delivery efficiency of UCTT-PC4 (RGD-) and UCTTD-PC4 (RGD+) by flow cytometry. (c) Statistical diagram of delivery efficiency in Capan-1 cells ($n = 3$, $**p < 0.01$).

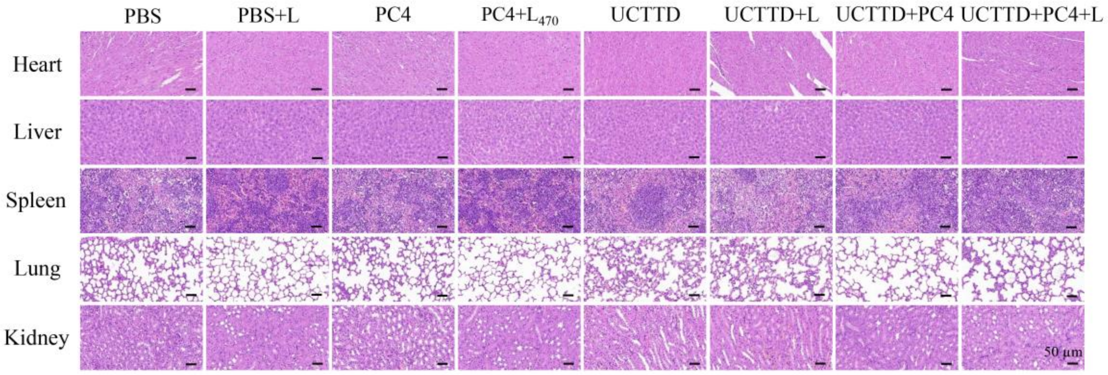

Figure S6. H&E staining images of major organs sections (heart, liver, spleen, lung, and kidney) of tumor-bearing mice from different groups after 14 days of different treatments. L_{470} represents 470 nm laser at power density of 0.08 W/cm^2 for 10 min, L represents 808 nm laser at power density of 1.2 W/cm^2 for 10 min. All scale bars are 50 μm.

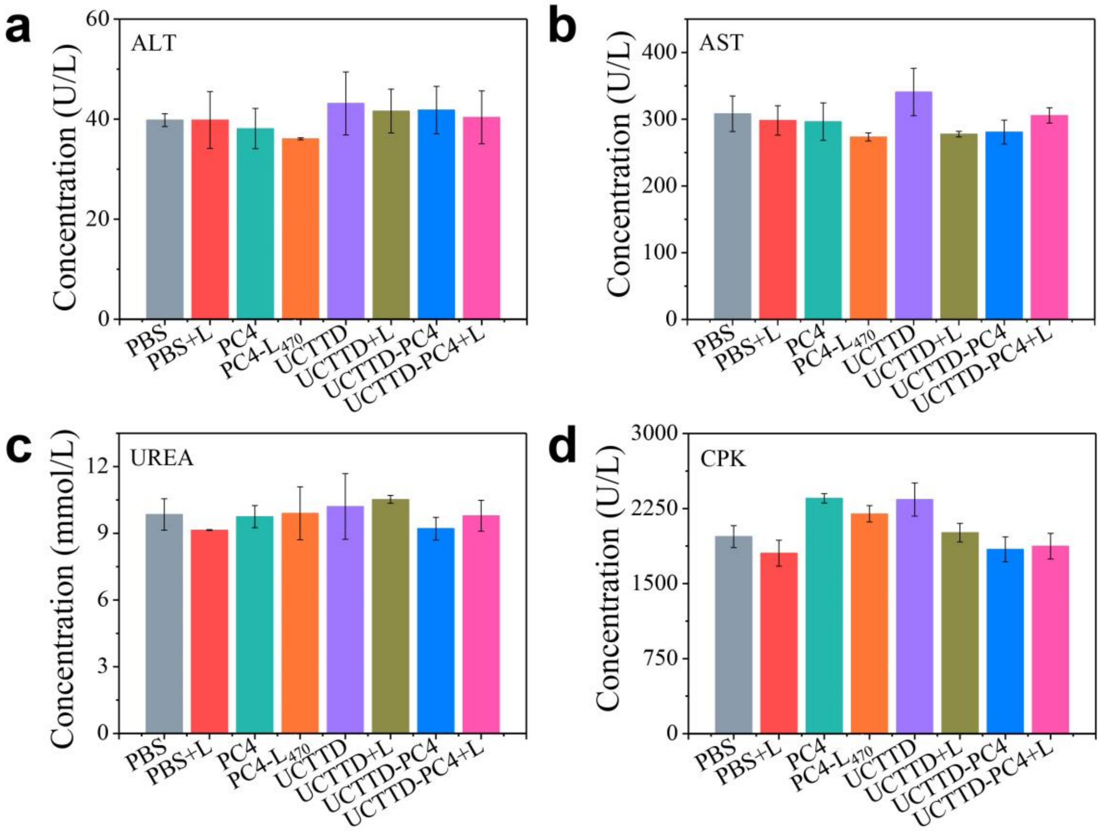

Figure S7. Blood biochemical parameters of Capan-1 tumor-bearing female BALB/c-nu mice upon different treatments for 14 days (n = 5). (a) The alanine aminotransferase (ALT), (b) aspartate aminotransferase (AST), (c) UREA, and (d) creatine phosphokinase (CPK) were evaluated through serum samples of the mice. Data are presented as means ± SD. L_{470} represents 470 nm laser at power density of 0.08 W/cm^2 for 10 min, L represents 808 nm laser at power density of 1.2 W/cm^2 for 10 min.

References

[1] D. M. Liu, X. X. Xu, F. Wang, J. J. Zhou, C. Mi, L. X. Zhang, Y. Q. Lu, C. S. Ma, E. Goldys, J. Lin, D. Y. Jin, *J. Mater. Chem. C* **2016**, *4*, 9227.

[2] H. Ejima, J. J. Richardson, K. Liang, J. P. Best, M. P. van Koeverden, G. K. Such, J. W. Cui, F. Caruso, *Science* **2013**, *341*, 154.

[3] X. N. Jia, W. G. Xu, Z. K. Ye, Y. L. Wang, Q. Dong, E. K. Wang, D. Li, J. Wang, *Small* **2020**, *16*, 2003707.
